# Gene-agnostic therapeutic approaches for inherited retinal degenerations

**DOI:** 10.3389/fnmol.2022.1068185

**Published:** 2023-01-09

**Authors:** Molly C. John, Joel Quinn, Monica L. Hu, Jasmina Cehajic-Kapetanovic, Kanmin Xue

**Affiliations:** ^1^Nuffield Laboratory of Ophthalmology, Nuffield Department of Clinical Neurosciences, University of Oxford, Oxford, United Kingdom; ^2^Oxford Eye Hospital, Oxford University Hospitals NHS Foundation Trust, Oxford, United Kingdom

**Keywords:** retina - medical therapies, inherited retinal degeneration, gene-independent, cellular reprogramming, stem cells, optogenetics, immune modulation

## Abstract

Inherited retinal diseases (IRDs) are associated with mutations in over 250 genes and represent a major cause of irreversible blindness worldwide. While gene augmentation or gene editing therapies could address the underlying genetic mutations in a small subset of patients, their utility remains limited by the great genetic heterogeneity of IRDs and the costs of developing individualised therapies. Gene-agnostic therapeutic approaches target common pathogenic pathways that drive retinal degeneration or provide functional rescue of vision independent of the genetic cause, thus offering potential clinical benefits to all IRD patients. Here, we review the key gene-agnostic approaches, including retinal cell reprogramming and replacement, neurotrophic support, immune modulation and optogenetics. The relative benefits and limitations of these strategies and the timing of clinical interventions are discussed.

## Introduction

### Inherited retinal diseases

**Figure 1:**
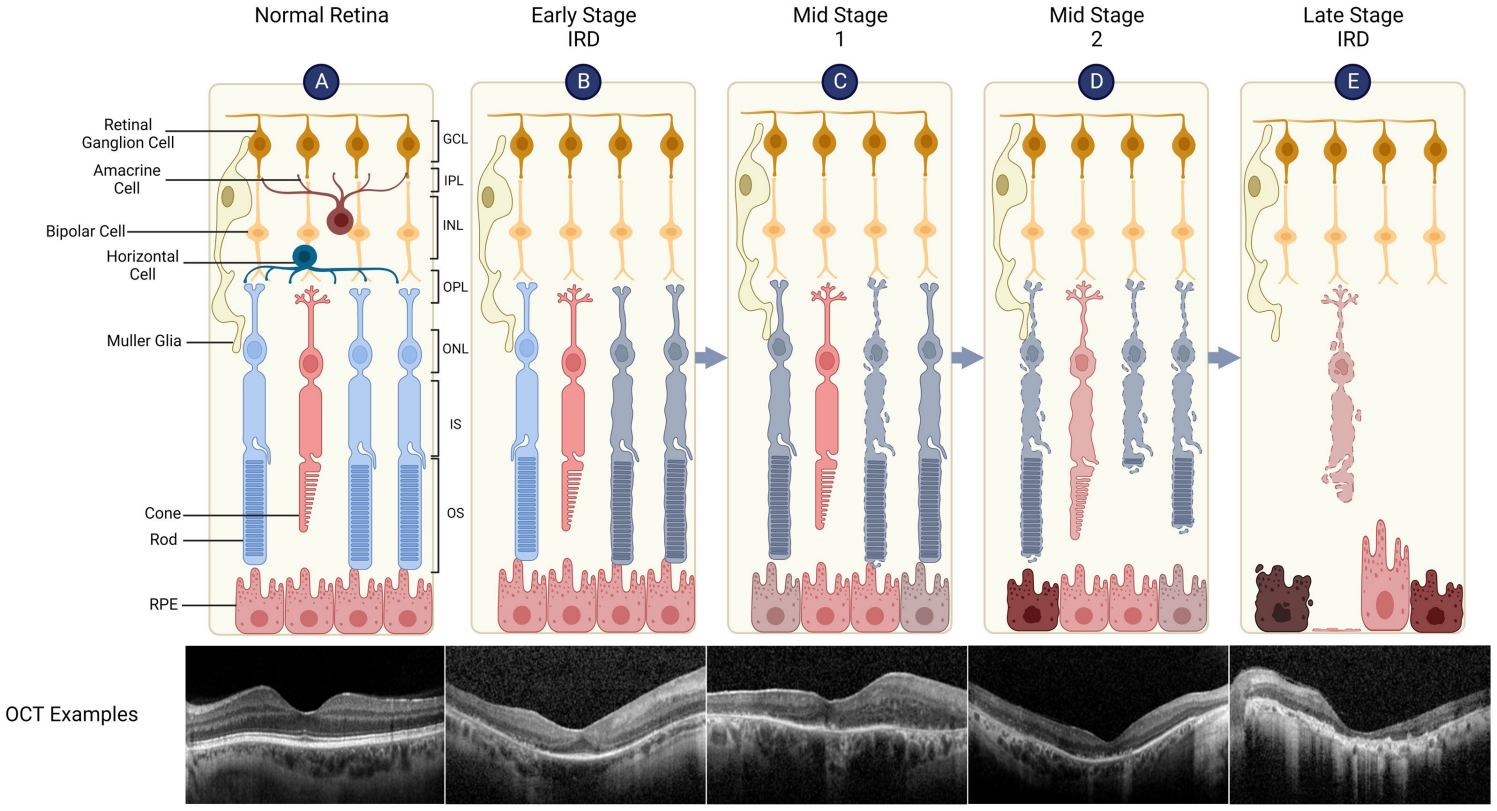
Retinal structure and degeneration in inherited retinal diseases (IRDs). The retina has a laminar structure consisting of distinct cell types **(A)**. The neural retina consists of the ganglion cell layer (GCL, containing the cell bodies of retinal ganglion cells), inner plexiform layer (IPL), inner nuclear layer (INL - bipolar cell bodies), outer plexiform layer (OPL), outer nuclear layer (ONL - photoreceptor cell bodies), inner segments (IS) and outer segments (OS). The retinal pigment epithelium (RPE) supports the metabolism of overlying photoreceptors, is attached to the Bruch’s membrane/choroid and forms outer blood-retinal barrier. A representative spectral domain optical coherence tomography (OCT) of the retina is shown which demonstrates normal retinal layers. **(B)** Early Stage IRD, such as retinitis pigmentosa, is typically characterised by dysfunction and degeneration of rod, which can be seen as peripheral outer retinal thinning on the OCT (note that parafoveal architecture is relatively preserved). **(C)** Retinal degeneration progresses to Mid Stage 1 where cone function (day light vision) remains relatively intact while rod function (night vision) is severely impaired. The OCT shows widespread disruption of the ellipsoid line which represents IS/OS junctions. RPE thinning can also be seen. **(D)** Mid Stage 2 sees cone degeneration with shortened OS and loss of rods. **(E)** In Late Stage (or end stage) IRD, there is complete loss of photoreceptors while inner retinal layers remain relatively preserved. OCT shows complete outer retinal atrophy with areas of RPE hypertrophy which correspond to bone spicules seen clinically.

Inherited Retinal Diseases (IRDs) are a genetically and phenotypically heterogenous group of diseases affecting the retina (Figure 1), the neural tissue responsible for visual function. IRDs are responsible for the majority of sight impairment in the working age population. Despite their classification as rare diseases, IRDs affect one in 4000 people or over 2 million individuals worldwide, causing a large healthcare burden and diminished quality of life for the affected individuals. A UK cost-of-illness study found IRDs were estimated to cost £523.3 million in 2019, of which £196.1 million represented additional wellbeing costs (Deloitte, 2022). Until recently no treatment options were available for IRDs.

Based on clinical patterns of outer retinal cell loss, IRDs have been categorised into rod-cone dystrophies, cone-rod dystrophies, chorioretinal degenerations and macular degenerations, though there is significant overlap between these classic phenotypes. To date, at least 250 genes have been identified as carrying causal mutations for IRDs (RetNet),^1^ including autosomal dominant, autosomal recessive, X-linked and mitochondrial inheritance patterns (Sahel et al., 2015; Gill et al., 2019). Rods, cones or retinal pigment epithelium (RPE) cells may be primarily affected dependent on the causal gene, leading to secondary degeneration of the other outer retinal cell types and eventual blindness. The most prevalent forms of IRDs include retinitis pigmentosa (RP), Stargardt disease, Leber congenital amaurosis (LCA) which causes childhood-onset sight loss, and syndromic IRDs most notably Usher syndrome, a ciliopathy in which photoreceptor degeneration is associated with hearing loss. Additionally, whilst not a typical IRD, age-related macular degeneration (AMD) has been shown to be associated with genetic polymorphisms which give rise to significantly increased disease risk. Thus, as with IRDs, AMD may be amenable to gene therapy and broader gene-agnostic approaches aiming to slow down retinal degeneration.

### Limitations of current gene therapies/approaches

Increased understanding of the genetic basis of IRDs by high-throughput sequencing has driven the development of gene or mutation-specific therapies for these previously untreatable diseases.

Adeno-associated viral (AAV) vector-mediated retinal gene therapies for IRDs have gained momentum in recent years, culminating in the approval of voretigene neparvovec (Luxturna), a gene augmentation therapy for bi-allelic *RPE65*-associated LCA. In this case, AAV-mediated gene delivery is highly attractive for an autosomal recessive disease, given the limited immunogenicity when injected into the subretinal space and rapid functional improvement from restoration of the visual cycle. Numerous other gene-specific therapies are under development or clinical trial. These range from gene augmentation, to CRISPR/Cas9-based genome/mRNA editing, to antisense oligonucleotide (ASO) approaches, which have been reviewed elsewhere (Kumaran et al., 2018; Quinn et al., 2021; Xue et al., 2021; Fenner et al., 2022).

Despite advancements in this field, gene-specific therapeutic approaches are limited by the high level of genetic heterogeneity amongst IRDs and difficulties in targeting dominant negative mutations. Gene-specific approaches also tend to benefit patients primarily in the earlier stages of disease, with intervention at a late stage when retinal degeneration has past ‘a point of no return’ (e.g., rod loss causing secondary cone degeneration) yielding worse results (Botto et al., 2022). AAV vectors have been chosen for transgene delivery for their tropism to retinal cells, non-mutagenic nature, and relative low immunogenicity, but are limited to a cargo capacity of ~4.7 kb which precludes delivery of many large IRD-related transgenes. Dual AAV delivery of *ABCA4* for Stargardt disease has been explored but efficacy is significantly limited by the need for co-transduction of target cells (Trapani et al., 2015; McClements et al., 2020a). Recently, viral vector-delivered CRISPR/Cas-based gene editing approaches have significantly broadened the scope of retinal gene therapy. Editing of genomic DNA focusing on IRD mutation hotspots or mRNA to partially restore normal protein production are under development (Dooley et al., 2018; Garanto, 2019; Fry et al., 2020). Despite their huge potential, gene editing therapeutics need to mitigate the risks of off-target mutations, immune responses to microbial-derived proteins (e.g., Cas9), and PAM site restrictions at specific disease target sequences (Wang et al., 2019; Uddin et al., 2020).

Whilst offering theoretical potential to treat many sub-groups of IRD patients, the development of many customised gene or mutation-specific therapies is practically challenging and tend to be associated with unsustainable costs (Orkin and Reilly, 2016).

### Gene-agnostic approaches for IRDs

Gene-agnostic (or gene independent) approaches for IRDs would be effective regardless of the specific gene defect thus providing the potential to treat a wide range of IRD patients. These therapeutic strategies are rapidly evolving, spanning from retinal cell reprogramming to immunomodulation, neuroprotection and optogenetics. These interventions generally target common pathways underlying photoreceptor death in retinal degenerations to circumvent the impacts of deleterious mutations. Separate to these are bioengineering solutions which are applicable in end-stage disease, such as the epiretinal Argus II retinal prostheses (Second Sight Medical Products, Inc., Sylmar, CA), subretinal Retinal Implant (Retinal Implant AG, Reutlingen, Germany) and suprachoroidal retinal implant (Bionic Vision Technologies, Australia). However development of these electronic prostheses falls outside the scope of this review and has been discussed elsewhere (Cehajic-Kapetanovic et al., 2022). In this review, we will discuss the main biological gene-agnostic strategies currently under development, and the challenges such therapies face, whilst offering insight into the future of IRD treatment.

## Retinal cell reprogramming

The potential to directly convert cell types *in vivo* to replenish target cells lost in disease is an exciting prospect in regenerative medicine, and in the treatment of retinal degeneration.

### Protective reprogramming in rod degenerations

Rod-cone dystrophies (RCDs) represent the largest fraction of IRDs, encompassing heterogeneous retinitis pigmentosa which account for 40% of all IRD cases. Patients with RCDs initially present with nyctalopia (night blindness) resulting from loss of rod photoreceptors responsible for scotopic vision. Progressive rod degeneration causes constriction of visual field, eventually leading to decrease in central visual acuity due to secondary cone death. The primary loss of rods in RCDs is believed to result from their high metabolic activity required to sustain continuous outer segment turnover and their dependence on the RPE, increasing susceptibility to IRD-causal mutations (Lin et al., 2015; Kwon and Freeman, 2020). Loss of rods is thought to increase oxidative stress on cones, reduce trophic support (e.g., *via* rod-derived cone viability factor), and release proinflammatory intracellular content (Brunet et al., 2022). While cone death may be a secondary event in RCDs, the loss of central vision has the greatest impact on the patient’s quality of life. Therefore, strategies to reduce the sensitivity of rods to metabolic stress or preserve cone function represent attractive gene-agnostic therapeutic avenues to combat RCDs.

**Figure 2:**
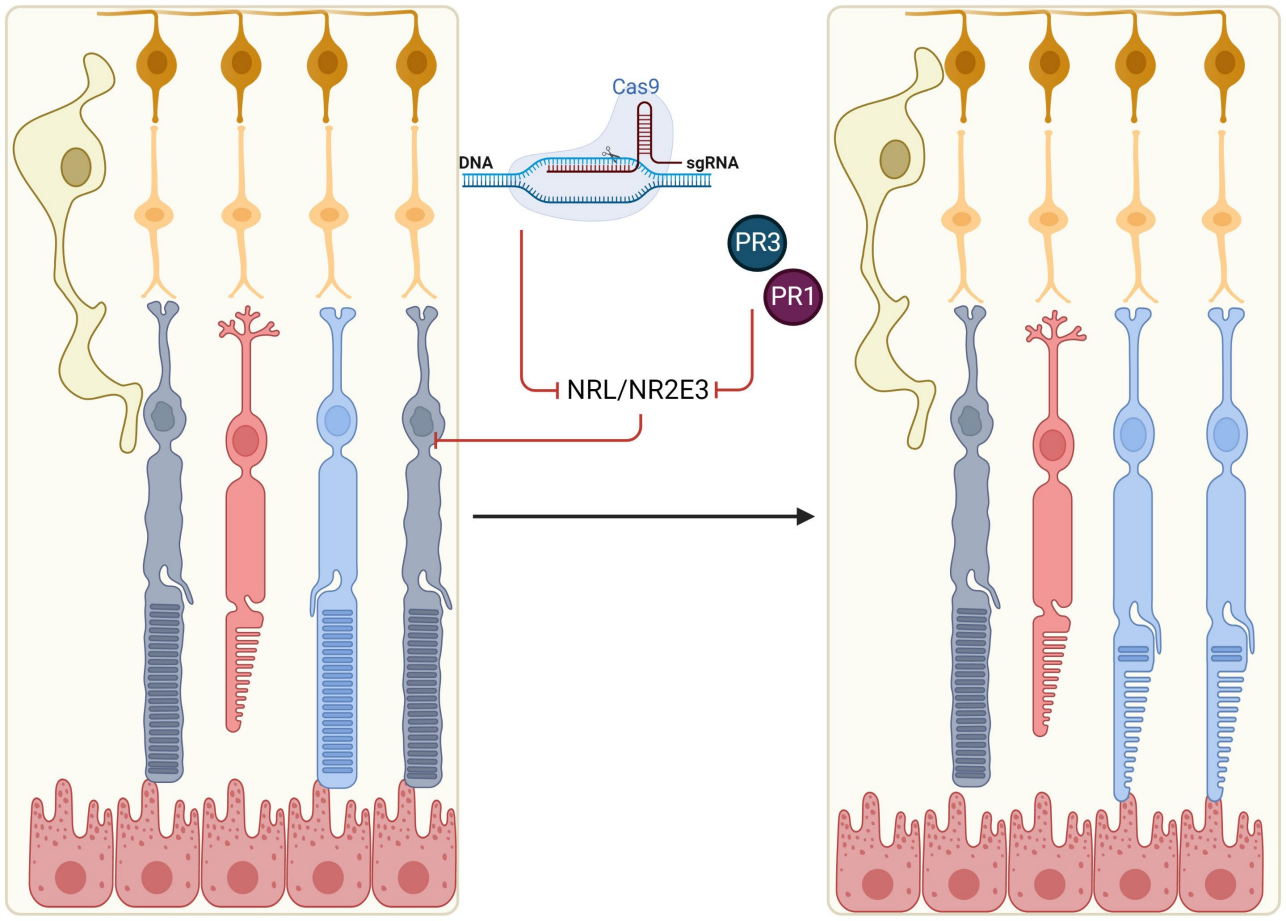
*In situ* reprogramming of rod photoreceptors. Rods may be reprogrammed in early stage IRDs to generate ‘pseudocones’ through manipulation of the NRL and NR2E3 transcription factors which normally determine rod fate (e.g., by CRISPR-mediated gene knockout or molecular inhibitors, PR1 and PR3). Pseudocones confer resistance against rod-specific gene mutations, thus slowing the rate of retinal degeneration.

One potential therapeutic approach is to induce trans-differentiation of mature rods towards a cone-like state, thus preserving light sensitivity whilst reducing risk of cell death (Figure 2). Developments in this area have predominantly focused on manipulation of the neural retina leucine zipper (NRL) and NR2E3 rod-differentiation transcription regulators. Expression of NRL in photoreceptor precursors induces transcription of the orphan nuclear receptor, NR2E3, which appears to suppress transcription of a range of cone-specific genes, thus committing NRL-positive cells to rod fate (Oh et al., 2008; Swaroop et al., 2010).

### Targeting NRL

Montana et al. (2013) first demonstrated the potential of photoreceptor reprogramming through the use of a floxed *Nrl* allele in *Rd1* and *Rho-/-* mice (Montana et al., 2013). Knockout (KO) of *Nrl* in the adult mouse resulted in alterations of gene expression patterns in the treated rods, upregulation of key cone-specific genes, as well as ultrastructural changes to cellular morphology. Crucially, the *Rho-/-* model showed preservation of rod cell bodies and outer segments past P90 when rod degeneration is normally complete in the untreated model, as well as preservation of cones and electroretinograms (ERGs) with increased photopic b-waves (indicative of enhanced cone response). Following this proof-of-concept, multiple groups have demonstrated similar results through CRISPR/Cas9-mediated knockout of *Nrl* (Yu et al., 2017; Zhu et al., 2017). Dual AAV strategies with vectors independently expressing small guide RNA (sgRNA) and Cas9 endonuclease were used to knockout the *Nrl* gene in mouse models of RP. An increase in cone-specific gene expression was seen coupled to a decline in rod-specific transcription, preservation of outer nuclear layer (ONL) thickness, and increase in photopic ERG b-wave responses. Notably, Yu et al. (2017) demonstrated *in vivo* effects of *Nrl* knockout in three different RP models of differing genetic backgrounds, with Zhu et al. (2017) testing one further model, indicating its potential as a gene-agnostic treatment for RCDs. An alternative strategy utilising CRISPR/Cas9-based gene repression approach demonstrated *Nrl* knockdown in the *rd10* mouse, preserved ONL thickness and improved visual function as determined by increased visual acuity by optokinetic nystagmus (Moreno et al., 2018). Moreno et al. also reported an AAV-split-Cas9 system with tetracycline response element (TRE) inducible expression of the Cas9 C-terminal, forming a ‘hit and run’ approach to CRISPR-induced NRL repression. Both systems demonstrate strategies to limit Cas9 nuclease activity and reduce the risk of off-target effects.

As well as mouse models, human retinal organoids derived from *NRL*^−/−^ embryonic stem cells demonstrated a shift in photoreceptor differentiation characterised by a lack of rod markers and increase in S-opsin positive cells (Cuevas et al., 2021). This *in vitro* data confirms the central role of *NRL* in human rod differentiation and points to potential cross-species applicability of the mouse results. However, unlike germline knockout which causes complete switching of retinal progenitor cell fate, knocking out *NRL* in the mature human retina may lead to partial conversion of rods to ‘pseudocones’ (or ‘cods’) (Mears et al., 2001). Notably, no significant upregulation of cone opsin expression was seen in the partially converted cells. This was likely due to established epigenetic modifications in mature rods as *Nrl* knockout did not alter methylation patterns at either the *Rho* or *Opn1sw* loci (Montana et al., 2013). Partial photoreceptor reprogramming may allow maintenance of normal retinal architecture, avoiding the formation of rosettes in the ONL as seen in the germline *NRL* knockout mouse (Mears et al., 2001; Roger et al., 2012).

### Targeting NR2E3

Acting immediately downstream of NRL, NR2E3 is also another transcription factor of interest in rod reprogramming. Inhibition of NR2E3 may be preferable by causing less drastic changes to cellular physiology whilst conferring similar therapeutic effects (Moore et al., 2020; Kolesnikov et al., 2022). *Nr2e3* knockout in *Rho*^−/−^ mice crossed with *Nr2e3*-deficient *rd7* strain showed similar phenotype as *Nrl* knockouts, with increased survival of rods and cones, and preservation of cone function up to 6 months as measured by ERG (Kolesnikov et al., 2022 – published abstract). Similar results were previously reported by Zhu et al. (2017) using a CRISPR knockout approach on *Nr2e3.*

Nr2e3 repression has also been achieved with small molecular drugs. Screening for molecular inhibitors of Nr2e3 in primary murine retinal cell cultures identified photoregulin 1 (PR1) which could reduce *Rho* expression (Nakamura et al., 2016). Administration of PR1 *in vivo* during retinal development demonstrated modulation of rod-specific gene expression and upregulation of a subset of cone-specific genes, though the data suggested that PR1 may also act by inhibiting Nrl. PR1 treated *Rho^P23H^* and *Pde6b^rd1^* retinae showed preservation of photoreceptors and ONL thickness (Nakamura et al., 2016). Further studies by the group to improve upon PR1’s pharmacological properties led to a structurally unrelated compound, photoregulin 3 (PR3). PR3 was shown to prevent photoreceptor loss, and preserve both scotopic and photopic ERG function in the *Rho^P23H^* mouse (Nakamura et al., 2017). These promising results in mice hold promise for potential translation of these drugs to clinical applications.

Contrary to aforementioned studies indicating the benefits of Nr2e3 suppression in retinitis pigmentosa, AAV8-mediated augmentation of *Nr2e3* at P0 was reported to improve photoreceptor survival and ERG responses in mouse models of IRDs (Li et al., 2021). The apparent similarity in effects of *Nr2e3* overexpression and suppression was attributed to differences in the timing of interventions, with Nr2e3 induced alteration of gene expression profiles of differentiating rods at birth counteracting the effects of the IRD mutations (Moore et al., 2020; Li et al., 2021).

Nevertheless, the prospect of protective rod reprogramming presents an intriguing gene-agnostic therapeutic approach for slowing disease progression in a range of rod-cone disorders. While there have been a number of proof-of-concept studies, questions remain regarding the mechanism of action. Downregulation of some rod-specific genes, such as rhodopsin, has been postulated to reduce metabolic stress, particularly in dominant negative IRD mutations (Athanasiou et al., 2018). Alternatively, there may be a secondary increase in neuroprotective or trophic factor expression that improve photoreceptor survival, though Yu et al. (2017) saw no changes to the expression of the rod-derived cone viability factors, *RdCVF* and *RdCVF2*. Another question relates to the long-term effects of *NRL* knockout. Germline loss-of-function mutations in *NR2E3* and to a lesser extent *NRL* have been associated with enhanced S-cone syndrome (ESCS) with variable retinal abnormalities (Wright et al., 2004; Audo et al., 2008). Deleterious effects have not been seen in the studies that manipulated these genes in the mature retina up to 6 month (Montana et al., 2013; Yu et al., 2017). This may be related to more limited impact on gene expression in the mature retina due to established DNA methylation patterns.

### Reprogramming Müller glia for retinal restoration

**Figure 3:**
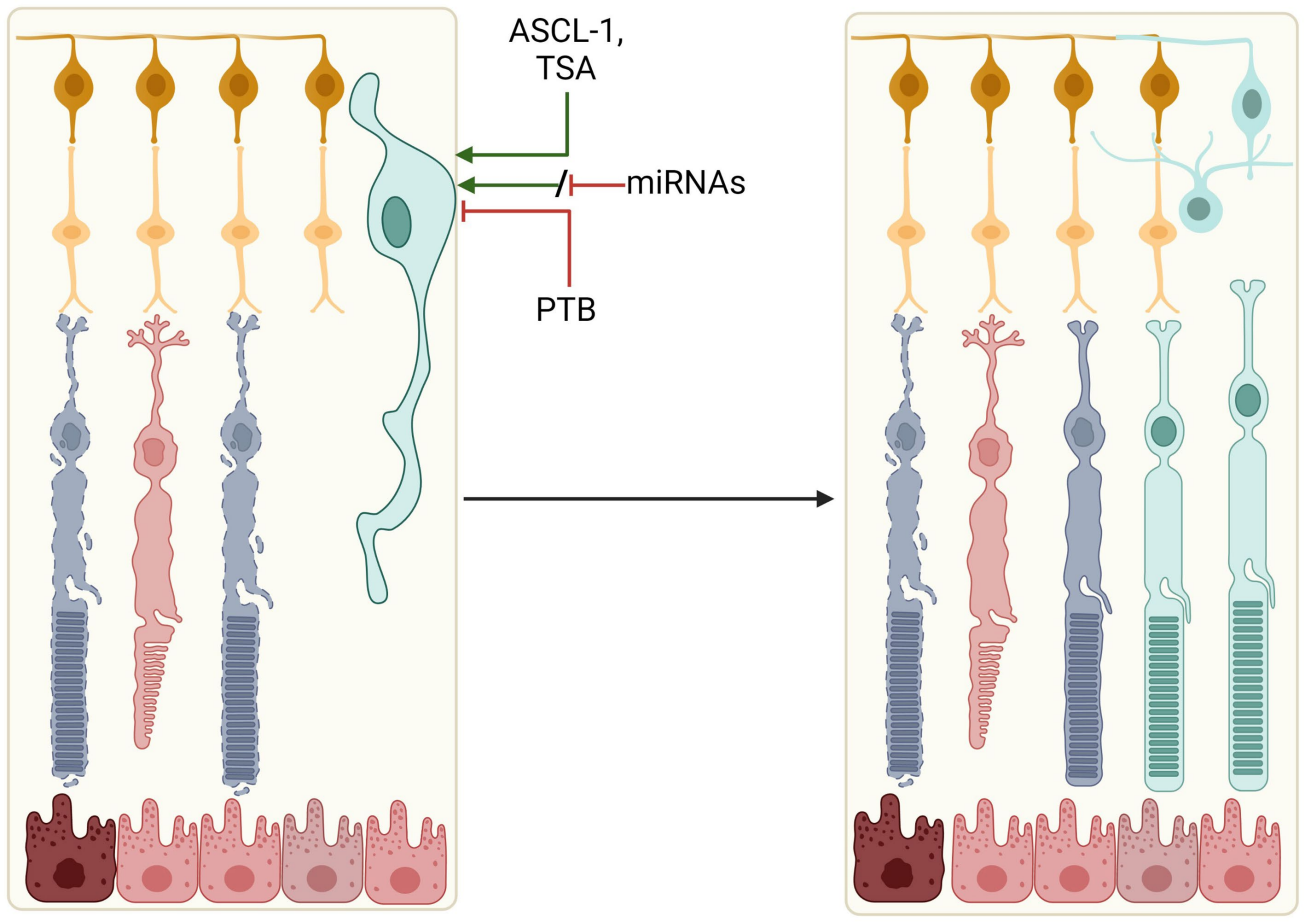
Reprogramming M*ü*ller glia to retinal cells. Manipulation of key signalling factors (ASCL-1, PTB, or miRNAs) could induce expansion and differentiation of resident retinal M*ü*ller glia into a number of retinal cell types. These include photoreceptors, amacrine cells and ganglion cells, which could replace lost and degenerating cells in the retina.

Separate to rod-conversion are strategies focused on Müller glia. Müller glia constitute the primary glial cell type of the retina, providing structural support and aiding in the maintenance of retinal homeostasis (Goldman, 2014). In zebrafish, Müller glia can adopt stem cell-like characteristics and differentiate into photoreceptor progenitor cells following retinal trauma in a process termed ‘reactive gliosis’, thus raising interest in exploiting Müller glia for mammalian retinal regeneration (Powell et al., 2016; Thomas et al., 2016). Regenerative reactive gliosis does not naturally occur in the mammalian retina despite Müller glia displaying an injury response (Dyer and Cepko, 2000; Karl et al., 2008), but attempts are being made to induce their differentiation and expansion into various retinal neuronal cell types (Figure 3).

Core signalling factors identified in zebrafish, including the ASCL1/Let-7, Wnt/β-Catenin, Notch and JAK/STAT pathways, have been manipulated in mammalian cells, demonstrating the relative plasticity of mammalian Müller glia and their potential for neural regeneration (Lawrence et al., 2007; Osakada et al., 2007; del Debbio et al., 2010; Giannelli et al., 2011; Singhal et al., 2012; Angbohang et al., 2016; Jorstad et al., 2020; Todd et al., 2020, 2021; Campbell et al., 2022). Notably, *in-vivo* studies of Achaete-scute homologue 1 (ASCL-1) overexpression were found to promote neuronal differentiation of Müller glia in the retinae of young mice following retinal damage, with evidence of amacrine cell, bipolar cell and rod-like photoreceptor generation. However, such a response was lacking in adult mice, positing the theory that fixed epigenetic DNA modifications and chromatin structures restricted the regenerative potential (Ueki et al., 2015). ASCL-1 expression in adult mice in combination with a histone deacetylase inhibitor TSA helped to overcome this limitation, inducing Müller glia differentiation (Jorstad et al., 2017).

Altering methylation patterns in mature retinal ganglion cells to promote axonal regeneration has also been investigated. Lu et al. (2020) demonstrated that viral vector-mediated overexpression of a subset of Yamanaka factors, *Oct4*, *Sox2*, and *Klf4*, in murine RGCs restored ‘youthful’ methylation patterns and promoted axonal regeneration following optic nerve crush injury (Lu et al., 2020). This poses an interesting approach of epigenetic reprogramming, as opposed to or in combination with genetic manipulation, as a mechanism to promote tissue repair.

Yao et al. (2018) reported the ability to generate rods *via* Müller glia, demonstrating *in vivo* reprogramming and restoration of visual responses in an IRD mouse model (*Gnat1^rd17^Gnat2^cpfl3^*; Yao et al., 2018). Here reprogramming was dependent on a two-step activation of Müller glia *via* the Wnt/β-catenin pathway, followed by supplementation of photoreceptor and rod-specific transcription factors, *Crx* and *Nrl* (Yao et al., 2018).

A CRISPR/CasRx-mediated approach for knocking down polypyrimidine tract-binding protein (*Ptbp1*) has been reported to induce Müller glia to retinal ganglion cell conversion and restore visual function *in vivo* (Zhou et al., 2020). This approach benefits from achieving results through a single target knockdown, as opposed to the manipulation of multiple signalling and pluripotent factors. However, it introduces the risk of off-target mRNA cleavage by CasRx. PTB has also been targeted *via* shRNAs and antisense oligonucleotides (ASOs), demonstrating the possibility of converting glial to neuronal retinal cell types using a number of potentially clinically relevant approaches (Fu et al., 2020; Qian et al., 2020; Maimon et al., 2021).

An alternative target for the reprogramming of Müller glia are MicroRNAs (miRNAs), conserved short (~22 nt) RNA sequences that have been shown to play a role in the regulation of gene expression and in the regeneration of the zebrafish retina and mammalian Müller glia activation (Wohl et al., 2017; O’Brien et al., 2018; Kittelmann and McGregor, 2019; Wohl et al., 2019; Konar et al., 2021; Kang et al., 2021). Numerous individual miRNAs have been interrogated for their role in Müller glia-mediated retinal degeneration (Konar et al., 2021). The miRNA *let-7* was shown to maintain the differentiated Müller glia state in zebrafish, with reduction in *let-7* level post injury allowing for derepression of a number of regeneration-associated factors and dedifferentiation and Müller glia expansion (Ramachandran et al., 2010). Numerous miRNAs were found to be differentially expressed in zebrafish MG-derived retinal regeneration, including miR-203, miR-7, miR-27, and miR-31 (Rajaram et al., 2014). miR-203 downregulation was demonstrated to be required for zebrafish regeneration, with artificial maintenance blocking retinal repair (Rajaram et al., 2014). Such findings suggest derepression of regeneration *via* miRNAs may prove possible without the requirement to supplement or overexpress numerous signalling and pluripotent factors.

Recent studies have also begun to shed light on the interplay between Müller glia, their regenerative potential and the immune microenvironment. Microglia have been shown to play a key role in the induction of Müller glia in the zebrafish retina, whilst prolonged inflammation results in suppression of the regenerative process (White et al., 2017). The impact of inflammation has been shown to be injury-context dependent, and suggests a future role for immunomodulation alongside regenerative medicine approaches (Zhou et al., 2022).

Despite promising results in recent years (Ueki et al., 2015; Wohl et al., 2017; Yao et al., 2018; Lu et al., 2020), potential clinical application of *in situ* Müller glia reprogramming still face several challenges. Primary of these is the need to convert Müller glia to retinal neurones that correctly and stably integrate into the existing neuronal circuit. Whilst visual function gains seen in several studies would suggest some degree of cell integration, they were limited in duration. Hoang et al. (2022) showed that conditional heterozygous and homozygous *Ptbp1* mutants gave no glia-to-neuron conversion when assessed with lineage tracing and single cell RNA-seq analysis (Hoang et al., 2022). This has raised questions about the stringency of studies reporting direct glia-to-neuron conversion, and the requirement for rigorous lineage tracing (i.e., transcriptomic profiling) of reprogrammed cells *in vivo* (Wang and Zhang, 2022; Xie and Chen, 2022).

Taken together, mammalian Müller glia retain a certain level of plasticity, and may be manipulated to differentiate into retinal neuronal cell types *in vivo*. The ability of the reprogrammed cells to integrate into the neuronal circuit remains to be seen, which will determine the therapeutic viability of this approach for retinal repair.

## Retinal cell replacement therapies

**Figure 4:**
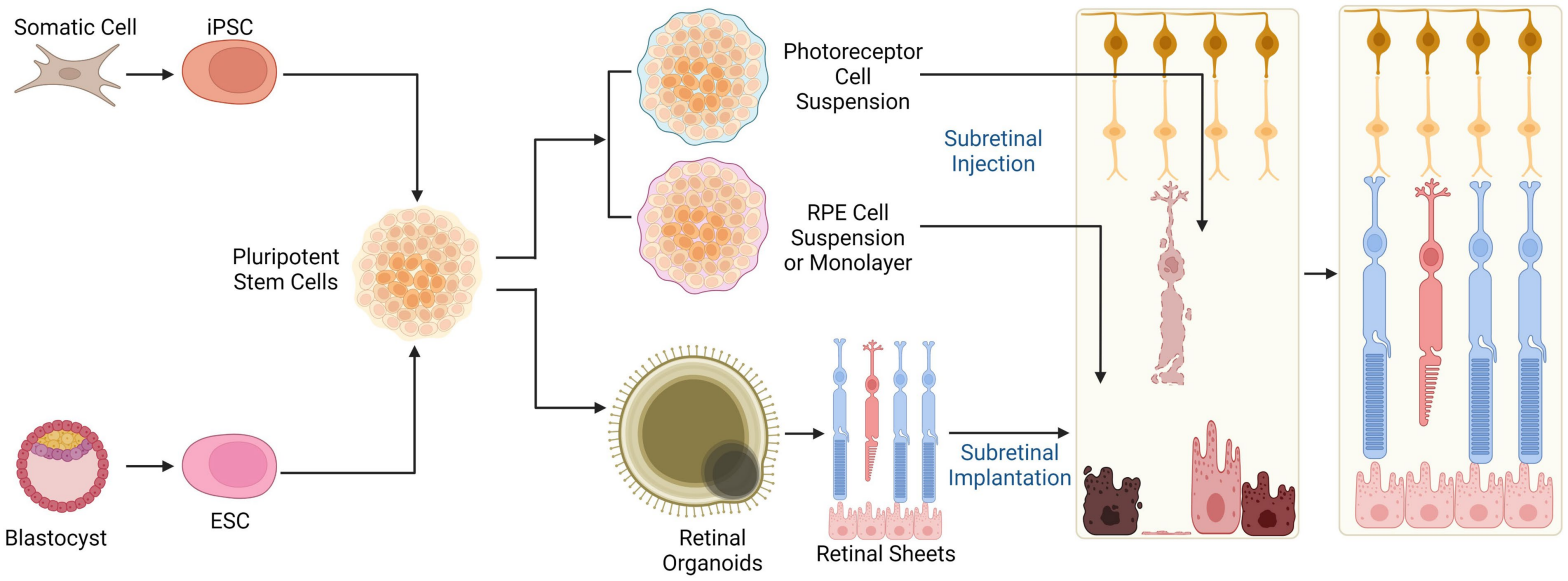
Cell therapies for retinal degenerations. Allogeneic stem cell-based cell replacement strategies rely on induced pluripotent stem cells (iPSCs) or embryonic stem cells (ESCs) which can be differentiated into photoreceptor or RPE cell suspensions, sheets, or retinal organoids for transplantation. The donor cells or tissue replace lost photoreceptors and RPE in late stage IRDs. Subretinal injection of cell suspensions allows direct contact between donor cells and the surviving neuronal cells in the retina but can result in disorganised engraftment. Subretinal implantation of structured retinal sheet helps to retain anatomical organisation, which may facilitate appropriate cellular differentiation but is surgically challenging.

Separate to approaches aiming to reprogram endogenous retinal cells are transplantation of exogenously derived retinal cells (chiefly RPE and photoreceptor precursors) with the aim to replace damaged or dysfunctional cell types in IRDs. Retinal cell transplantation strategies have been explored over several decades (Jayakody et al., 2015; Gasparini et al., 2019; Cehajic-Kapetanovic et al., 2022). Recent advances have been aided by developments in stem cell biology, through the reprogramming of embryonic stem cells (ESCs) and induced pluripotent stem cells (iPSCs) (Takahashi and Yamanaka, 2006; Figure 4). ESCs and iPSCs have been successfully differentiated into numerous retinal cell types *in vitro*, which can be harvested at virtually every developmental stage for the purpose of cell therapy. The ability to generate ESC/iPSC-derived functional retinal organoids in culture further expands treatment options (Eiraku et al., 2011; Nakano et al., 2012; Reichman et al., 2014; Zhong et al., 2014). A number of retinal cell therapies have now reached clinical trial.

### Photoreceptor transplantation

Photoreceptor replacement to restore visual function requires *de novo* synaptic connections between the transplanted cells and retinal interneurons as well as appropriate interactions between the photoreceptors and underlying RPE.

Early attempts of photoreceptor transplantation used foetal retinal tissue with some transient functional rescue in AMD and RP patients (Das et al., 1999; Radtke et al., 2008). The use of such tissue raises complex ethical dilemmas and concerns about immunological rejection, and has been superseded by other approaches. Later mouse studies utilised purified photoreceptor suspensions from dissociated retinae, tissue-derived stem cells or rod-committed photoreceptor precursor cells (Tomita et al., 2002; MacLaren et al., 2006; Gust and Reh, 2011; Pearson et al., 2012). Notably, Maclaren et al. demonstrated integration of mouse donor *Nrl*-expressing rod precursors, suggesting that early rod lineage commitment may be an optimal time for integration with host retinal cells (MacLaren et al., 2006). Subsequently, transplanted *Nrl*-GFP-tagged mouse rod precursors were shown to establish synaptic connections with host bipolar and horizontal cells and restore scotopic visual function in *Gnat1-/-* mice which have non-functioning rods (Pearson et al., 2012). Similarly, human ESC/iPSC derived photoreceptor precursors have been transplanted into *Crx deficient* mice (a model of LCA) with restoration of light responses (Lamba et al., 2009, 2010). In contrast to rod transplantation, cone replacement has been more challenging. Flow-sorted embryonic (but not postnatal) *Crx*-expressing photoreceptor precursors have been transplanted into *rd8* and *Gucy2e*^−/−^ mice, and found to differentiate into both cones and rods (Lakowski et al., 2010). Approaches utilising photoreceptor cells taken from *Nrl*^−/−^ mice, in which all rods become converted to cone-like cells, showed integration and photopic response restoration in Cpfl1 mice though integration rates were low (<1%) (Santos-Ferreira et al., 2015). The results of these studies were later revaluated in light of evidence for ‘cytoplasmic fusion’ whereby intracellular material from labelled donor cells (e.g., fluorescent protein) in the subretinal space may be transferred to host photoreceptors thus creating a false impression of cell integration (Santos-Ferreira et al., 2016; Singh et al., 2016). While this casts doubt over the feasibility of direct photoreceptor replacement therapy, it presents the new possibility of *in vivo* cytoplasmic transfer as a mode of host photoreceptor rescue in RP.

Recent promising advances have been made with Ribeiro et al. (2021) reporting successful transplantation of human PSC-derived cones into *rd1* mouse retina. Synapse formation was observed between donor photoreceptors and host bipolar cells, with restoration of light-evoked ERG responses and behaviours (Ribeiro et al., 2021). Crucially, the inclusion of non-functional CNGB3 (c.1148delC) hiPSC cones as a transplant control indicate the rescue effect seen is unlikely to result from cytoplasmic transfer. CNGB3 deficient cells showed survival and maturation post transplantation but no rescue of retinal or visual function was observed despite such cells containing the same array of potentially transferrable molecules as functional hPSC-cones.

The delivery of stem cell suspensions has progressed to clinical trial in the treatment of retinitis pigmentosa. A phase 1/2 trial by jCyte (NCT02320812) delivered hRPCs, with results indicating that the cells were well tolerated in patients. Phase 2b trials (NCT03073733) demonstrated some efficacy of hRPC delivery in high-dose patients, as determined by mean change in BCVA from baseline to month 12. A phase 1/2 dose escalation study conducted by ReNeuron is also ongoing, assessing safety, tolerability and preliminary efficacy of a subretinal hRPC injection (NCT02464436) with results expected soon.

Alongside delivery of retinal cell suspensions is the possibility of delivering structured cell sheets derived from 3D tissue culture techniques, which may improve graft survival and function compared with cell suspensions (Gasparini et al., 2019). Subretinal transplantation of day 11-24 ESC and iPSC-derived retinal sheets into *rd1* mice, which have lost all photoreceptors through rapidly progressive RP, led to formation of photoreceptors with outer segments and signs of host-graft synaptic connections (Assawachananont et al., 2014; Mandai et al., 2017). Transplantation of human ESC-derived retinal sheet into the subretinal place of chemical or laser-induced outer nuclear layer-depleted retinae in non-human primates (NHPs) demonstrated graft photoreceptor maturation and survival up to 5 months, but no functional visual improvement was detected by ERG (Shirai et al., 2016). In contrast, human ESC-retina transplanted in immunodeficient rat model of severe RP (*rho S334ter-3* nude) did lead to improved optokinetic and ERG responses (McLelland et al., 2018). Despite the therapeutic promise of retinal sheet transplantation, there remain challenges to be addressed. Subretinally transplanted retinal sheets often show formation of IS/OS-containing rosettes which are thought to represent mal-arranged rod outer segments that would be expected to reduce the functionality of the graft (Assawachananont et al., 2014; Mandai et al., 2017; Thomas et al., 2021). Transplantation of multilaminar retinal sheets containing outer and inner nuclear layers may lead to duplication of the inner nuclear layer in advanced RP thus preventing correct synaptic connections. Separation of 3D cultured stem cell-derived retinal layers for transplantation would be technically challenging, although recent developments utilising 3D engineered micro-scaffolds may offer an alternative [reviewed in: (Cehajic-Kapetanovic et al., 2022)]. The first clinical trial of human iPSC-derived retinal sheets in patients with advanced RP patients began in 2020 (Japan registry of clinical trials ID: jRCTa05020002). Technical limitation of graft size (to approximately 1 mm diameter) will need to be taken into account when interpreting the functional effects seen but trial outcomes are yet to be reported.

### RPE transplantation

Transplantation of RPE is primarily aimed at treating AMD and Stargardt disease where loss of RPE is a primary pathogenic driver. Similar to photoreceptor transplantation, early studies demonstrated feasibility of transplanting RPE from foetal retinal tissue or translocation of autologous RPE sheets from the periphery to the macula (Algvere et al., 1999; van Zeeburg et al., 2012). RPE cells were first generated *in vitro* from ESCs in 2001, and were subsequently shown to enhance survival of host photoreceptors when subretinally transplanted into Royal College of Surgeons (RCS) rat and mouse models (Kawasaki et al., 2002; Haruta et al., 2004). Later, Li et al. reported improvement in visual function in iPSC-RPE treated *rd12* mice by ERG testing (Li et al., 2012). These proof-of-concept studies rapidly translated into clinical trials of RPE transplantation in AMD and IRD patients.

There are two prevailing RPE cell therapy products: an RPE sheet (with or without membrane scaffold) or cell suspension (Maeda et al., 2022; Seraly et al., 2022). While donor RPE cells do not need to integrate into the retinal neural network, correct polarisation of the RPE monolayer is essential for function. The latter is a potential advantage of RPE sheet transplantation, but insertion of a sheet under the retina is technically challenging and require parallel development of novel surgical techniques (da Cruz et al., 2018).

Interestingly, transplantation of combined iPSC-RPE and iPSC-retinal progenitor cells was found to provide better conservation of ONL and ERG responses compared with RPE or RPC therapy alone (Salas et al., 2021). An interesting study delivering iPSC-derived ‘retinal cells’ concurrently exhibiting both RPE and photoreceptor characteristics also demonstrated preservation of visual function in a *Pde6b* knockout rat model as assessed by ERG. The donor cells survived up to 9 months in the retina, and retained characteristics of both cell types, though no evidence of retinal neural connection was seen at the transplant site and the cells did not form normal retinal lamination (Yang et al., 2021). A ‘co-graft’ technique has also been trialled in RCS rats consisting of a hESC retinal organoid-derived retinal progenitor sheet combined with RPE cells using a bio-adhesive (gelatin, growth factor-reduced matrigel, and medium viscosity (MVG) alginate) showing co-graft survival, photoreceptor differentiation and integration, and visual function improvement by optokinetic testing (Thomas et al., 2021). Since RPE and photoreceptor loss are often closely correlated in IRDs, preservation of visual function may be better achieved by replacing both cell types. Therefore these studies offer interesting insights into future clinical treatment and novel transplantation approaches.

Alongside clinical trials of RPE transplantation in AMD patients, assessment of subretinal transplantation of MA09-hRPE hESC-derived cell line (Klimanskaya et al., 2006) in Stargardt disease patients showed graft survival and safety up to a median of 22 months, with some BCVA improvements in treated versus untreated eyes (Schwartz et al., 2015, 2016). Other trials conducted in Stargardts patients with hESC-RPE have also proved promising. Recent clinical data from a Phase1/2 trial of MA09-hRPE in 12 Stargardt patients demonstrated reasonable safety of subretinal RPE cell suspension injection, with no evidence of uncontrolled proliferation or severe immune response (Mehat et al., 2018). This study did however highlight the risks associated with higher cell doses with instances of focal retinal thinning with RPE hyperpigmentation and reduced sensitivity. A number of other clinical trials of RPE cell therapy are ongoing with a recent summary provided by Sanie-Jahromi and Nowroozzadeh (2022).

Preclinical studies have suggested reasonable functional benefit from the use of these cell-based therapies, with some results from clinical trials showing such interventions are well-tolerated, and do not appear associated with either tumorigenesis or increased inflammation in patients (Schwartz et al., 2015, 2016; Mehat et al., 2018). Transplantation therapy through the use of ESC/iPSC-derived retinal sheets may prove particularly beneficial, allowing for restoration of both the photoreceptors and the RPE in a more stable manner (Assawachananont et al., 2014; Shirai et al., 2016; Mandai et al., 2017; McLelland et al., 2018; Gasparini et al., 2019).

## Neuroprotection and control of inflammation in early IRDs

Accumulating evidence points to a significant role for dysfunctional metabolism, neurotrophic support and inflammation in the progression of secondary cone death in IRDs.

### Delivery of neurotrophic factors

Delivery of neurotrophic factors to the degenerating retina has shown beneficial effects on photoreceptor survival in several models of retinal degeneration. One such factor is ciliary neurotrophic factor (CNTF), where non-viral delivery alleviated photoreceptor loss in the *rd1*, nervous (nr/nr) and *Rho*^Q344ter^ mouse models of retinal degeneration (LaVail et al., 1998), *Rdy* feline model of retinal atrophy (Chong et al., 1999), and *rcd1* canine model of retinitis pigmentosa (RP) (Tao et al., 2002). Similarly, viral supplementation of CNTF improved photoreceptor survival in *Rho*^-/-^ mouse model of RP (Liang et al., 2001; Lipinski et al., 2015). However, there is conflicting evidence against the beneficial effects of CNTF supplementation therapy. In the *rd2* mouse, AAV-mediated murine CNTF supplementation did reduce photoreceptor loss but had negative effects on visual function as measured by ERG (Schlichtenbrede et al., 2003). The loss of visual function was later shown to be dose-dependent (Buch et al., 2006). Studies in *Peripherin/rds* mouse showed that AAV-delivered CNTF can suppress cone opsin expression thus decreasing light sensitivity despite positive effects on photoreceptor survival (Rhee et al., 2007). These findings were ultimately recapitulated in humans, where delivery of CNTF by encapsulated intraocular implants did not lead to any visual improvement in RP patients despite preservation of ONL compared with sham-treated eyes (Birch et al., 2013).

Glial cell derived neurotrophic factor (GDNF) is another neurotrophic factor which has shown success in slowing photoreceptor loss in the *rd1* mouse (Frasson et al., 1999), *Rho*^S334ter^ rat (Sanftner et al., 2001), *rd2* mouse and RCS rat (Buch et al., 2006). This appears to be an indirect effect caused by increased GDNF signalling in retinal glial cells, as the GDNF receptor was only found to be expressed on Müller glia in porcine retina. Subsequent experiments showed that GDNF upregulated production of basic fibroblast growth factor (*FGF-2 gene encoding* FGF-β) in cultured Müller glia, and that FGF-β has a positive effect on photoreceptor survival *in vitro* (Hauck et al., 2006). Intravitreal injection of FGF-β in combination with minocycline, a microglial activation inhibitor, also had moderate beneficial effects on photoreceptor number and morphology in the P23H-1 and RCS rat models of retinal degeneration (di Pierdomenico et al., 2018). Despite promising pre-clinical results, no data is currently available on whether GDNF could provide therapeutic benefit in human retinal degenerations.

### Manipulation of photoreceptor metabolism

An alternative strategy is to target dysfunctional metabolism that occurs during retinal degenerations. Photoreceptors demand large amounts of glucose, of which 80-96% is converted to lactate *via* aerobic glycolysis and is required for anabolic processes and outer segment maintenance (Hurley et al., 2015; Chinchore et al., 2017). Based on gene expression data from 4 mouse models of RP, and the observation that insulin provided a significant protective effect on cones, it was suggested that cone death results in part from glucose deprivation (Punzo et al., 2009). This pathogenic retinal glucose shortage may be due to the retention of glucose in the RPE, caused by loss of contact with phosphatidylserine on rod outer segments (Wang et al., 2016, 2019).

Metabolic reprogramming of photoreceptors has had success in producing beneficial effects on vision in animal models of retinal degeneration. Genetic or shRNA-mediated ablation of *Sirt6*, a glycolytic repressor, reprogrammed photoreceptors to shuttle glucose towards anaerobic metabolism and improved visual function in the Pde6b^H620Q^ mouse model of retinal degeneration (Zhang et al., 2016).

Recently, AAV8-mediated delivery of Txnip, a thioredoxin-interacting protein, improved cone number in the rd1, rd10 and Rho-/- models of RP (Xue et al., 2021). Interestingly, the Txnip(C247S) mutant, which has abrogated binding to thioredoxin, further improved cone survival. Subsequent experiments suggested that Txnip shifts cone metabolism towards catabolism of non-glucose substrates (e.g., lactate, ketone bodies and fatty acids), increasing the supply of ATP available for anabolic processes in the absencBe of glucose. Delivery of Txnip variants with reduced GLUT1 downregulation activity to the RPE also improved cone survival, possibly by reducing glucose dependence in the RPE, thus allowing more glucose to be delivered to cones (Wang et al., 2016; Xue et al., 2021).

Rod-derived Cone Viability Factor (RdCVF) is a neurotrophic factor secreted by rods that also impacts cone metabolism. The *Nxnl1* as the gene encodes a truncated thioredoxin-like protein, RdCVF, and a longer isoform with a full thioredoxin-like fold, RdCVFL (Fintz et al., 2003; Léveillard et al., 2004). RdCVF stimulates glucose uptake and aerobic glycolysis in cones, while RdCVFL appears to perform an anti-oxidative function (Aït-Ali et al., 2015; Clérin et al., 2020).

Subretinal delivery of RdCVF protein to Rho^P23H^ rats resulted in an increase in cone number and function, as measured by (ERG) (Yang et al., 2009). Furthermore, intravitreal delivery of an AAV7m8-scCAG-RdCVF construct rescued cone survival and function in both rd10 and RhoP23H mice (Byrne et al., 2015). Co-delivery of AAV-packaged RdCVF and RdCVFL also showed a slight improvement over RdCVF alone in the rd10 model. This has led to the development of SPVN06, an AAV gene therapy encoding both RdCVF and RdCVFL, which is currently in the latter stages of pre-clinical validation (Lorget et al., 2022). SPVN06 is also being trialled in tandem with SPVN20, a potassium ion efflux channel protein that is opened by endogenous opsin-associated G-proteins, allowing signal transduction in dysfunctional cones still expressing cone opsins and arrestin (Simon et al., 2022). The combination of trophic support factors and optogenetic modalities may enhance cone rescue when compared with either alone.

### Control of retinal inflammation

Inflammation is also becoming appreciated as an important factor in the progression of retinal degenerations, and may present opportunities for therapeutic intervention. Neuronal damage and death during IRDs can lead to release of damage-associated molecular patterns (DAMPs), which are recognised by innate immune receptors on microglia. This can trigger microglial activation and release of pro-inflammatory molecules, particularly Tumour Necrosis Factor-α (TNF-α), Interferon Gamma (IFN-γ), IL-6, IL-1α, IL-1β, CCL2 (MCP-1) and CCL8 (MCP-2) (Yoshida et al., 2013). These molecules aid recruitment of circulating leukocytes to the degenerating retina, resulting in inflammation. Clinical evidence from patients with (RP) shows immune cell infiltration in the anterior chamber and elevated pro-inflammatory cytokine and chemokine levels in the vitreous (Newsome and Michels, 1988; Yoshida et al., 2013). Animal models of RP also provide supporting evidence that the innate immune response and inflammation play a role in disease, with multiple therapies targeting the immune system showing beneficial effects in retinal degeneration (Sudharsan et al., 2017; Guadagni et al., 2019; Table 1). Thus, potential treatment strategies include cytokine inhibition, and suppressing microglial activation and subsequent cytokine release [reviewed in; Akhtar-Schäfer et al., 2018; Olivares-González et al., 2021)].

**Table 1:** Targeting retinal inflammation as a gene-agnostic approach to treating retinal degenerations.

Target	Function	Intervention	Animal model	Reference
TNF-α	Pro-inflammatory cytokine	Antagonistic antibody (adalimumab), systemic or local (intravitreal)	*Rd10* mouse	Martínez-Fernández De La Cámara et al. (2015)
			*Rd10* mouse	Olivares-González et al. (2020)
		Genetic knockdown	Rho^T17M^ mouse	Rana et al. (2017)
NLRP3	Inflammasome activation (*via* IL-1β/IL-18) in response to cell damage	Genetic knockout and inhibition (N-acetylcysteine)	Rho^P23H^ rat	Viringipurampeer et al. (2016)
IL-1R	Pro-inflammatory cytokine receptor	Inhibitory antibody (Kineret) and peptide (rytvela)	Blue light-induced retinal damage in mouse	Dabouz et al. (2020)
CCR2	Chemokine receptor on monocytic phagocytes	Genetic knockout	*Rd10* mouse	Guo et al. (2012)
		Genetic knockout	Blue light-induced retinal damage in mouse	Hu et al. (2016)
P2X7	ATP receptor involved in ATP-triggered apoptosis	Genetic knockout and antagonist (A438079)	Optic nerve crush in mouse	Nadal-Nicolás et al. (2016)
A2AR	Adenosine A_2A_ receptor	Antagonist (SCH58261)	Diabetic retinopathy in mouse	Aires et al. (2019)
		Antagonist (ZM241385)	Retinal detachment in mouse	Gao et al. (2020)
		Antagonist (SCH58261)	Light-induced retinal degeneration, rat	Soliño et al. (2022)
SIGLEC-11	Primate-specific receptor for polysialic acid (a neuronal self-recognition molecular pattern)	Polysialic acid with average degree of polymerisation of 20 (polySia avDP20)	Laser injury, human SIGLEC-11 knock-in mouse	Karlstetter et al. (2017)
TLR2/TLR4	Innate immune pattern-recognition receptors	Minocycline	*Rd10* mouse	Scholz et al. (2015b)
CCL3	Chemokine	Genetic knockout	*Mertk^-/-^* mouse	Kohno et al., (2014)
TSPO	Mitochondrial cholesterol transporter	Molecular agonist (XBD173)	Light-induced retinal degeneration, mouse	Scholz et al. (2015a)

Cytokine and chemokine inhibition has shown beneficial effects in several animal models of retinal degeneration. Genetic deficiency of the CCL2/CCR2 axis prevents mononuclear phagocyte recruitment to the retina, and improved vision in the *rd10* mouse (Guo et al., 2012) and a mouse model of chronic blue light-induced damage (Hu et al., 2016).

TNF-α is a key pro-inflammatory cytokine in multiple retinal disorders, and TNF-α blocking antibodies have been shown to slow retinal degeneration in the rd10 mouse (Martínez-Fernández De La Cámara et al., 2015; Olivares-González et al., 2020). Genetic knockdown of TNF-α also had beneficial effects on photoreceptor survival in the Rho^T17M^ mouse (Rana et al., 2017).

The NLRP3 inflammasome is involved in the proteolytic processing and release of IL-1β and IL-18, which are drivers of photoreceptor death (Charles-Messance et al., 2020). Inhibition of the NLRP3 inflammasome had beneficial effects on visual function and photoreceptor survival in the RhoP23H rat model of RP (Viringipurampeer et al., 2016). Additionally, inhibition of the of the IL-1β receptor, IL-1R, resulted in suppression of inflammation, improvements in visual function and photoreceptor survival in a model of blue light-induced damage (Dabouz et al., 2020). Given that IL-1β is also significantly increased in the vitreous humour of patients with RP compared to those with idiopathic epiretinal membrane (Yoshida et al., 2013), this inflammatory process may play an important role in the progression of degenerative disease and presents an interesting point for therapeutic intervention.

Blocking DAMP recognition and signalling in microglia is another anti-inflammatory strategy that may have neuroprotective effects. For example, ATP release from damaged neuronal cells is recognised by purinergic receptors on microglia and induces the release of pro-inflammatory cytokines and NLRP3 inflammasome activation (Viringipurampeer et al., 2016). The ATP receptor P2X7 has been postulated as a target for intervention, as P2X7-deficient mice showed delayed retinal neuron loss in an optic nerve crush model of retinal ganglion cell loss (Nadal-Nicolás et al., 2016), while a P2X7 antagonist delayed retinal ganglion cell loss in the same model. Furthermore, patients with P2RX7 mutations that abolish innate phagocytosis show increased risk of age related macular degeneration, indicating an important role for the receptor in retinal health (Gu et al., 2013). However, careful consideration of the role of P2X7 in different disease contexts and selection of compounds for pharmacological modulation of its activity is required, as its biology remains incompletely understood (Savio et al., 2018).

Another target is the A2AR receptor, which binds to adenosine and leads to the production of NO and cytokine release. A2AR blockade (LaVail et al., 1998), Rdy feline model of retinal atrophy (Chong et al., 1999) and the rcd1 canine model of retinitis pigmentosa (RP) (Tao et al., 2002). Similarly, viral delivery of CNTF improved photoreceptor survival in the Rho-/- mouse model of RP (Liang et al., 2001; Lipinski et al., 2015), diabetic retinopathy (Aires et al., 2019) and retinal detachment (Gao et al., 2020).

Primate microglia express SIGLEC-11, which recognises poly-sialic acid caps on the neuronal glycocalyx, providing an inhibitory signal through an immunoreceptor tyrosine-based inhibitory motif (ITIM). Neuraminidases secreted by infiltrating immune cells cleave the poly-sialic acid caps, removing the microglial inhibitory signal and also enabling opsonization of the altered glycocalyx by C1q, which is recognised by microglial CR3 (Wang and Neumann, 2010). Mice expressing human SIGLEC-11 and treated with poly-sialic acid with an average degree of polymerisation of 20 (polySia-20) showed reduced mononuclear phagocyte activation and vascular leakage in a laser injury model (Karlstetter et al., 2017).

Microglial inhibition has also been achieved through the use of minocycline, a tetracycline derivative. Minocycline inhibits TLR2 and TLR4 signalling through NF-κB in microglia, and has protected against retinal degeneration in a light-damage model and the *rd10* mouse model of RP (Scholz et al., 2015b).

Providing a potential link between inflammation and dysfunctional metabolism, Conart et al. showed that vitreous samples from patients with retinal detachment had elevated levels of pro-inflammatory cytokines. In addition, using a mouse model of retinal detachment, they showed that infiltrating immune cells downregulate RdCVF production, and that either inhibiting immune cell infiltration with TSP1 or stimulating the insulin pathway can improve cone survival (Conart et al., 2020).

Several studies have also demonstrated the interplay between neuroprotective factors, metabolism and retinal inflammation through environmental manipulation (Barone et al., 2012; Dieguez et al., 2021). Environmental enrichment, defined as increasing visual stimuli, motor and social activities, resulted in upregulation of trophic factors, notably BNDF, preserving photoreceptors and visual function by ERG in the rd10 mouse and an induced AMD model (Barone et al., 2014; Dieguez et al., 2021) with indication of reduced retinal inflammation (Guadagni et al., 2019).

While dysfunctional metabolism and inflammation present many interesting avenues of investigation for gene agnostic treatments for IRDs, clinical data on agents targeting these potential disease mechanisms is limited. Future clinical trials with novel and existing agents will inform whether they can significantly improve visual outcomes in patients.

## Optogenetic restoration of visual function in advanced IRDs

**Figure 5:**
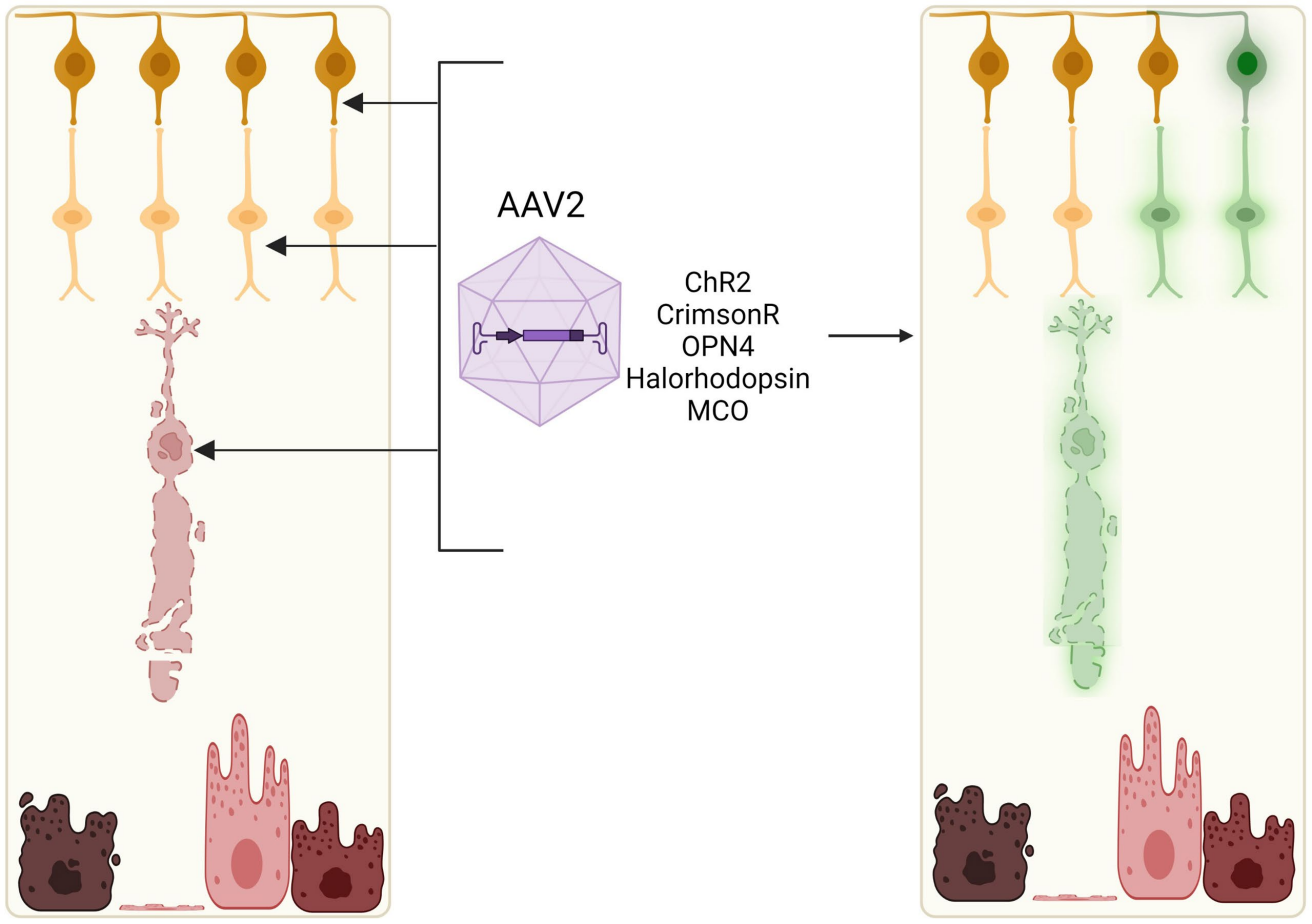
Optogenetic induction of light sensitivity in inner retinal cells. As bipolar and ganglion cell layers often remain relatively intact in retinal degenerations, light sensitivity may be induced in these neuronal cell types through viral vector-mediated delivery of opsins, e.g., channelrhodopsin-2 (ChR2), channelrhodopsin-CrimsonR, halorhodopsin, or multicharacteristic-opsin (MCO). Current clinical trials of optogenetic therapies utilise adeno-associated viral vectors which can efficiently transduce retinal ganglion cells, bipolar cells, and some remaining cone cell bodies *in vivo*.

Optogenetic therapy merges optical and genetic engineering approaches in order to introduce light-sensitive proteins into inner retinal neurons that are normally light insensitive. By targeting surviving cell types with intact neural circuitry, optogenetics offer an avenue to bypass degenerate photoreceptors in a gene-agnostic manner of restoring vision (McClements et al., 2020b; de Silva and Moore, 2022; Lindner et al., 2022). This can be achieved by delivering genes encoding opsins, which are light-sensitive transmembrane proteins of microbial or animal origin, to be expressed ectopically in the target retinal cell type (Figure 5).

Conventional retinal gene therapy generally aims to restore the function of a gene in cells where is it natively expressed, and thus requires that the target cell population (e.g., rod and cone photoreceptors) has survived to a sufficient number that is permissive to achieving functional rescue. In advanced IRDs that have progressed to significant, irreversible loss of the outer retina, this is not always a viable tactic. While structural and functional remodelling of the residual inner retina has been observed after photoreceptor death, the surviving neurons appear stable, active and receptive to input (Jones et al., 2003; Marc et al., 2003; Marc and Jones, 2003; Pfeiffer et al., 2020). Optogenetic therapy takes advantage of this remaining neural architecture for receiving visual stimuli. Following proof-of-concept in preclinical models, several investigational optogenetic therapies have progressed to clinical trials (NCT04919473; NCT02556736; NCT03326336; NCT04278131).

### Different types of opsins for optogenetic therapies

Opsins studied for optogenetic applications (Table 2) can be classed as microbial opsins (Type 1) or animal opsins (Type 2) (Simon et al., 2020). They vary in characteristics such as the mechanism of activation, the peak wavelength of light responded to, light sensitivity and response kinetics.

**Table 2:** Characteristics of key opsins used in optogenetic therapeutic studies.

Opsin	Origin	Type	Peak activation wavelength	Reference	Key preclinical or clinical studies
Channelrhodopsin 2 (ChR2)	Green alga *Chlamydomonas reinhardtii*	Microbial light-gated cation channel (depolarising)	460 nm	Nagel et al. (2003)	*rd1* mice (Bi et al., 2006; Zhang et al., 2009; Gaub et al., 2015; Macé et al., 2015)
					*rd1*, *rd10*, and *rd16* mice (Doroudchi et al., 2011) Royal College of Surgeons (RCS) rats (Tomita et al., 2010)
					NCT02556736
Calcium translocating channelrhodopsin (ChR–CatCh)	L132C mutant of ChR2 with enhanced Ca^2+^ permeability		474 nm	Kleinlogel et al. (2011)	Human retinal organoid (Garita-Hernandez et al., 2018)
					*rd1* mice (Cronin et al., 2014)
					Non-human primate (Chaffiol et al., 2017)
Red-light activated depolarising channelrhodopsin (ReaChR)	Engineered red-shifted ChR variant from Green alga *Volvox carteri*		590 nm	Lin et al. (2013)	Human retinal organoid (Garita-Hernandez et al., 2018)
					*rd1* mice (Sengupta et al., 2016)
ChrimsonR	Green alga *Chlamydomonas noctigama*		600 nm	Klapoetke et al. (2014)	Human retinal organoid (Garita-Hernandez et al., 2018)
					Non-human primates (McGregor et al., 2020; Gauvain et al., 2021)
					NCT03326336 (ref Sahel et al., 2021)
Chronos	Green alga *Stigeoclonium helveticum*		500 nm		NCT04278131
Enhanced halorhodopsins (eNpHR, eNpHR 2.0 and 3.0)	Engineered variants from archaebacterium *Natronomonas pharaonis*	Microbial light-gated chloride pump (hyperpolarising)	590 nm	Gradinaru et al. (2008)	Human retinal organoid (Garita-Hernandez et al., 2018)
				Gradinaru et al. (2010)	*rd1* mice (Busskamp et al., 2010)
					*Cpfl1/Rho*^−/−^ mice (Garita-Hernandez et al., 2019)
Jaws	Engineered crux-halorhodopsin from *Halobacterium salinarum* (strain Shark)		600 nm	Chuong et al. (2014)	Human retinal organoid (Garita-Hernandez et al., 2018)
					*rd1* mice Chuong et al. (2014)
					*Cpfl1/Rho*^-/-^ and *rd1* mice (Garita-Hernandez et al., 2019)
					Non-human primates (Khabou et al., 2018)
Rhodopsin (RHO)	Human	Vertebrate G-protein-coupled receptor	496 nm	–	*rd1* mice (Cehajic-Kapetanovic et al., 2015; Eleftheriou et al., 2017; Gaub et al., 2015; McClements et al., 2021)
Melanopsin (OPN4)	Human		480 nm	–	*rd1* mice (Lin et al., 2008; Liu et al., 2016; de Silva et al., 2017)
Medium wavelength cone opsin (MWC)	Human		531 nm	–	*rd1* mice (Berry et al., 2019; McClements et al., 2021)
Multicharacteristic opsin (MCO)	(Proprietary)	(Proprietary)	500 nm; ambient white light used	Wright et al. (2017)	*rd10* mice (Wright et al., 2017; Batabyal et al., 2021)
					NCT04919473
					NCT04945772
					NCT05417126

Microbial opsins, such as channelrhodopsin-2 (ChR2), halorhodopsin (NpHR) and their engineered variants, are light-activated ion channels or pumps that directly influence neuron polarisation on activation by facilitating ion flux. Channelrhodopsins act as cation channels that cause depolarisation, while halorhodopsins act as chloride pumps that cause hyperpolarisation. Thus, successful membrane expression of these opsins would not require any extra signalling cascade components to generate a response. One concern regarding the use of microbial opsins is the higher potential for triggering an immune response and inflammation. In addition, light sensitivity of microbial opsins is significantly less than animal opsins; for instance, the light stimulus required to obtain a response from channelrhodopsin-2 is 10^15^ photons cm^–2^ s^–1^, many orders of magnitude higher compared to cones (10^10^ photons cm^–2^ s^–1^) or rods (10^6^ photons cm^–2^ s^–1^) (Lagali et al., 2008). Such intensities are not usually encountered in normal lighting conditions, and thus require an adjunctive high energy light source for activation, raising concerns for light toxicity to the retina (Bi et al., 2006; Zhang et al., 2009; Doroudchi et al., 2011; Sengupta et al., 2016). Despite these challenges, microbial opsins have shown efficacy in preclinical models and progressed to clinical trials.

Animal opsins, such as rhodopsin and cone opsins, act as G protein-coupled receptors that when activated indirectly cause cell hyperpolarisation *via* the closure of cGMP-gated cation channels (McClements et al., 2021). Ectopic expression of human opsins in the retina is likely to be less immunogenic than microbial opsins. G protein signalling leads to greater signal amplification than stimulation of independent ion channels or pumps, enabling responses to be evoked at lower, more physiological light intensities than microbial opsins, and without raising concerns regarding light toxicity (Lindner et al., 2022).

### Preclinical developments

Progress since the first landmark *in vivo* optogenetics study was completed in *rd1* mice (Bi et al., 2006) has been rapid, with many preclinical studies successfully achieving improvements in visual function following opsin expression in mouse and non-human primate models (Table 1). Commonly used is the *rd1* mouse model, due to the rapid rod-cone degeneration phenotype it displays resulting from a *Pde6b* nonsense mutation emulating late-stage retinal degeneration (Carter-Daw et al., 1978). Multi-electrode array (MEA) recordings from retinal explants of treated animals provide a useful assessment of the kinetics and intensity of the electrical response to light stimulus, with spatiotemporally encoded visual responses attainable at the retinal level (Cehajic-Kapetanovic et al., 2015; Eleftheriou et al., 2017). Further propagation of signals to central brain areas has been shown through measuring responses from lateral geniculate nucleus – the first central synaptic region receiving information directly from retinla ganglion cells (Cehajic-Kapetanovic et al., 2015) and visual evoked potentials (Liu et al., 2019; Tabata et al., 2021) or changes in blood flow or oxygenation in the visual cortex associated with light stimulus (van Wyk et al., 2015; de Silva et al., 2017). Behavioural assessments, for example light avoidance behaviour, the optomotor response, visual context recognition tasks and visually evoked changes in locomotor activity (Cehajic-Kapetanovic et al., 2015; de Silva et al., 2017; Batabyal et al., 2021), are an important indicator of whether these changes convert into functionally useful vision.

Opsin genes are relatively small in size (~2 kb) and can easily be packaged within the AAV capsid limit, allowing this strategy to benefit from concurrent advances in AAV engineering and retinal transduction. Rational optimisation of *in vivo* optogene expression stems from selection of the AAV serotype (or capsid modification) and gene promoter (ubiquitous versus cell-specific; McClements et al., 2020b). While gene-specific therapies for IRDs tend to target RPE and outer retinal photoreceptors, optogenetic therapy approaches typically target distal neurons, i.e., ON bipolar cells (Lagali et al., 2008; Doroudchi et al., 2011; Cronin et al., 2014; Cehajic-Kapetanovic et al., 2015, van Wyk et al., 2015) and retinal ganglion cells (Cehajic-Kapetanovic et al., 2015; Sengupta et al., 2016; Berry et al., 2019). Surviving cone photoreceptors that have lost their outer segments also make a valid target (Busskamp et al., 2010). Various murine studies have achieved improvements in visual responses from ubiquitous opsin expression (Bi et al., 2006; Lin et al., 2008; Tomita et al., 2010; Cehajic-Kapetanovic et al., 2015; de Silva et al., 2017), whilst restricting expression to ON-bipolar cells achieved more varied and better-quality visual percepts (Cehajic-Kapetanovic et al., 2015). Given the complexity of retinal circuitry, a challenge remains to predict how conversion of downstream interneurons to photosensitive inputs will provide interpretable visual signals in humans. Results from clinical trials will thus be vital in assessing the visual experiences created by optogenetic transformation of these different retinal neuronal populations.

### Clinical trials

A number of investigational therapeutics have emerged in the optogenetics clinical pipeline. All current studies utilise recombinant AAV2 (or variant thereof) administered *via* intravitreal injection for optogene delivery. Thus far, investigators from only one trial have published a peer-reviewed report on results in the first patient (Sahel et al., 2021), and care must be taken in interpreting results from press releases published by the sponsoring companies of the remainder.

Nanoscope Therapeutics completed a phase 1/2 dose escalation trial (NCT04919473) in 11 RP patients using vMCO-010 (previous name vMCO-I), a proprietary multi-characteristic opsin (MCO) packaged in AAV2 and delivered intravitreally. MCO is activated by ambient light and thus does not require an external device. A press release reported dose-dependent improvements sustained at 1 year, with improvements in visual acuity, shape discrimination and mobility tests (Nanoscope Therapeutics, 2021). This optogene, which includes a fluorescent reporter, has continued to two phase 2B trials in RP (NCT04945772) and Stargardt (NCT05417126) patients.

A phase 1/2a dose-escalation study for an intravitreal injection delivering the channelrhodopsin-2 transgene using AAV2 (RST-001) run by RetroSense Therapeutics (NCT02556736) has recruited 14 RP patients and is ongoing after dosing the first patient in 2015 (RBV Capital, 2022). No serious adverse effects have been reported.

The PIONEER study (NCT03326336), led by Gensight Biologics, is investigating the combination of an intravitreally injected optogenetic vector with light-stimulating goggles. GS030-DP is an AAV2/7 m8 vector expressing the channelrhodopsin ChrimsonR fused with tdTomato. A neuromorphic camera in the goggles transforms real-world visual events (pixel-by-pixel changes in contrast) into monochromatic images that are projected onto the retina as 595 nm light pulses, a wavelength selected due to the peak sensitivity of ChrimsonR-tdTomato being around 590 nm (Sahel et al., 2021). Results from 84 weeks post-treatment of the first RP patient demonstrated an improvement from baseline perception of light to being able to perform simple visuomotor tasks [videos available in Supplementary data (Sahel et al., 2021)]. Visual training with the goggles commenced at 4.5 months post injection, a time point selected because ChrimsonR-tdTomato expression in foveal ganglion cells was observed to stabilise at 2-6 months post injection in nonhuman primates (Gauvain et al., 2021). Using a head-scanning strategy, the patient was able to perceive and locate objects on a white table only when using the goggles, and described looking for ‘vertical vibrations’ as a visual cue. Furthermore, object-related visual events (assessing the presence or absence of a black tumbler on a white table) corresponded with occipital alpha desynchronisation activity recorded on electroencephalography, providing evidence for propagation of retinal activity to the primary visual cortex. Qualitatively, the patient reported being able to identify daily objects (plate, mug, phone, furniture) while using the goggles. No evidence of intraocular inflammation or retinal anatomy changes were found, and there were no ocular or systemic adverse events. The use of tdTomato again represents a new frontier in allowing reporter constructs to be delivered into humans. The ChrimsonR-tdTomato fusion construct showed greater efficacy than ChrimsonR alone in non-human primates, and was believed to assist protein trafficking to the membrane (Gauvain et al., 2021). Another non-therapeutic function is to allow detection of successful expression of the opsin transgene for stimulation by the goggles, although the authors were unable to do so as red fluorescent probe detection with scanning laser ophthalmoscopy is not yet approved for clinical use (Sahel et al., 2021). Long-term data from reporter protein expression in the human retina will provide useful safety information.

Another modified channelrhodopsin, ChronosFP, is being studied in a phase 1/2 trial by Bionic Sight (NCT04278131) in combination with a ‘neural coding device’ (GlobeNewswire, 2021). A press release reporting results from the first four dosed patients observed improvements in light sensitivity and motion detection (GlobeNewswire, 2021).

In all, preliminary data emerging from clinical trials show promise for optogenetic therapy as a way of restoring light perception to the degenerate retina regardless of the causative mutation. Long term safety effects of microbial opsin expression in the retina will be important to monitor, as well as what potential advantages human opsin transgenes might offer (McClements et al., 2021) and what unique visual experiences can be achieved in patients seeing through altered retinal circuitry.

## Discussion and future perspectives

Increased understanding of the pathophysiology of retinal degeneration in IRDs has led to the development of numerous broadly applicable therapeutic approaches alongside mutation or gene-specific gene therapies. These gene-agnostic strategies offer the potential for treatment across the spectrum of IRDs, though the timing and mode of intervention should take into careful account the state of disease progression (Figure 1) or IRD subtype.

Rod-cone dystrophies are initially characterised by rod dysfunction due to deficiency of a specific protein or accumulation of mutant protein. *In situ* cellular reprogramming of rods aims to intervene at this early disease stage to prevent rod death and subsequent secondary photoreceptor loss, thus extending visual function. Key proof-of-concept and preclinical data supporting this therapeutic approach have been obtained (Montana et al., 2013; Yu et al., 2017; Zhu et al., 2017; Moreno et al., 2018), however clinical translation remains some way off, in part due to outstanding questions surrounding mechanistic details and effects on scotopic versus mesopic visual function. Clinical development is also likely to be tied to improvements in vector design and CRISPR technology, which would allow improved cellular targeting and efficiency of NRL/NR2E3 knockdown while minimising off-target effects (a key safety concern). To this end, the split-Cas9 *NRL* repression system developed by Moreno et al. offers a promising approach, inducing knockdown in a potentially reversible manner (Moreno et al., 2018). Alternatively, small molecular inhibitors of NR2E3 (Nakamura et al., 2017) may be delivered intravitreally to enable photoreceptor reprogramming without inducing any permanent genomic changes. Another clinical consideration is the potential impact of rod-to-pseudocone conversion on visual function and retinal structure. Based on patients with enhanced S-cone syndrome, the treatment may lead to nyctalopia, altered colour vision with increased sensitivity to blue light. These effects will have to be balanced against the benefits of slower IRD progression and prolonged visual function.

Another broadly applicable mode of intervention in early to mid-stage of IRDs is retinal immune modulation. Preclinical data has cemented the role of inflammation and break down of the blood-retinal barrier as a common pathogenic driver in retinal degenerations (Newsome and Michels, 1988; Yoshida et al., 2013; (Sudharsan et al., 2017; Guadagni et al., 2019; Brunet et al., 2022), and highlighted the potential for immune modulation to control disease progression (Martínez-Fernández De La Cámara et al., 2015; Scholz et al., 2015b; Olivares-González et al., 2020). Despite some promising data on agents that target pro-inflammatory cytokine and chemokine signalling, clinical data is currently limited and questions remain as to the timing and duration of immune interventions. While immune modulation would not address the fundamental genetic defect underlying the IRDs, it may prove to be an essential adjunct for slowing the rate of disease progression thus extending the treatment window for other targeted interventions. In addition, retinal inflammation is increasingly recognised as a limiting factor in viral vector-mediated retinal gene therapies in which vector-associated foreign antigens may activate innate immune responses (Chandler et al., 2021). Thus adjunctive immune modulation may help prevent deleterious inflammation and improve the clinical efficacy of retinal gene therapies.

Later stage rod-cone dystrophies are characterised by significant loss of rods associated with secondary degeneration of cones. Rescuing the remaining viable cones could potentially prolong central vision. Neuroprotective or metabolic modulation approaches that act to prevent cone degeneration, independent of rods, are potentially applicable to a wide range of RPs. Amongst a number of trophic factors investigated to date, RdCVF is perhaps the most promising candidate with strong preclinical data supporting its importance for cone survival (Byrne et al., 2015; Lorget et al., 2022). The results of clinical trial of SPVN06 and SPVN20 (by SparingVision) as a combination therapy to prevent cone loss and restore light-induced signal transduction in dysfunctional cones in mid to late stage RP is eagerly anticipated (Sparing Vision, 2022).

Due to irreversible loss of native photoreceptors in late stage IRDs, *in situ* reprogramming of retinal Müller glia towards photoreceptor-like cells represents a potential minimally invasive therapeutic strategy compared with cell transplantation. Whilst evidence suggest that mammalian Müller glia retain some level of plasticity and may be induced to differentiate into photoreceptors and ganglion cells (Ueki et al., 2015; Wohl et al., 2017; Yao et al., 2018; Lu et al., 2020), questions remain as to the completeness of the glia-to-neuronal conversion (Hoang et al., 2022). Moreover, the longevity of the Müller glia-derived neurons is unclear. To achieve clinically relevant levels of visual function improvement, a large proportion of Müller glia will need to undergo differentiation thus depleting the resident population of which provide structural and homeostatic support to the retina (including cones). Therefore, therapeutic Müller glia reprogramming may need to achieve a fine balance between over-conversion and under-conversion to achieve clinical feasibility. The interplay between Müller glia and other resident glial cell types, predominantly microglia, is also an area of interest, implicating immunomodulation as an potential adjunct to Müller glia reprogramming (White et al., 2017; Zhou et al., 2020).

The end stages of IRDs is associated with near complete outer retinal cell loss with RPE migration into the subretinal space and some neuronal remodelling, including dendritic reorganisation and cell migration, in the inner retina (Marc et al., 2003) Therefore, gene-specific therapies, which primarily target photoreceptors, are unsuitable for patients at this stage. Stem cell-based cell transplantation could potentially rescue visual function in advanced IRDs by reconstituting one or more cell types in the retina (Schwartz et al., 2015, 2016). However, there are a number of outstanding questions relating to the best methods for cell derivation, administration and integration. Sheet-based cell transplants may prove more stable, facilitating donor cell survival and anatomical integration compared with cell suspensions, but pose greater surgical challenges (Singh et al., 2020). Clinical trials to date have primarily used ESC-derived cells, demonstrating early efficacy and reassuringly low risk of tumorigenesis (Schwartz et al., 2015, 2016; Mehat et al., 2018). Derivation of retinal cells from iPSCs offer the possibility of autologous transplantation, which would significantly reduce the risk of immune rejection and requirement immunosuppression (Sugita et al., 2021). However, consideration must be made for the limitations of introducing cells which harbour the original disease-causing genetic mutations which could the donor cells to degenerate over time. Alternatively, *in vitro* correction of genetic mutations in patient-derived iPSCs would be technically possible using CRISPR-based approaches, but would create new logistical challenges relating to quality control of the donor cells (e.g., to rule out oncogenic off-target mutations) and regulatory approval (Bassuk et al., 2016; Burnight et al., 2017). Thus, currently allogenic HLA-matched ESC or iPSC-derived retinal cells represent the most practically viable gene-agnostic cell therapy options (Sugita et al., 2021). The establishment of GMP-compliant ESC/iPSC banks, standardised accelerated differentiation protocols, and improved surgical delivery techniques will help to address some of the key challenges for cell transplantation in the future (da Cruz et al., 2018).

An alternative approach to rescuing visual function in late stage IRD patients is through the use of optogenetic approaches, which has seen recent clinical proof-of-concept (Nanoscope Therapeutics, 2021; Sahel et al., 2021). Current optogenetic approaches using microbial opsins, whilst proving viable in early clinical trial, may provide limited light sensitivity thus necessitating the use of light signal amplification devices (e.g., customised camera and goggle system) which may dampen patient uptake. This limitation might be overcome using ‘evolved’ or engineered opsins with enhanced light sensitivity. However, potential immune responses to microbial or artificially engineered opsin proteins remain a safety concern in the long-term. Progress in bringing endogenous human opsins, which can function without supranatural lighting conditions and being theoretically non-immunogenic, to clinical trial will add to the optogenetics toolbox. In addition, understanding and supplementing the downstream opsin (G-protein coupled receptor) signal transduction pathway within the target inner retinal cells could further improve clinical performance of optogenetic therapies. In the future, optogenetic strategies may also be combined with other gene-agnostic approaches, such as neurotrophic factor supplementation (Lorget et al., 2022; Simon et al., 2022; Sparing Vision, 2022) or iPSC-derived photoreceptor precursor transplantation (Garita-Hernandez et al., 2019) to maximise clinical benefits.

## Conclusion

Gene-agnostic therapies offer the potential to treat a broad spectrum of IRDs irrespective of the underlying genetic mutation and across different disease stages. A number of promising gene-agnostic approaches are currently in preclinical and clinical development. Key to the future of IRD treatment is likely to be combined approaches which synergistically address a number of pathogenic drivers specific to the disease stage, including retinal cell loss, deficiency of neurotrophic support, breakdown of retinal immune privilege, as well as the primary genetic mutations.

## Author contributions

KX and JC-K: conception and funding. MJ, MH, and JQ: drafting. MJ: figures. All authors contributed to the article and approved the submitted version.

## Funding

This work was funded by the Wellcome Trust (KX, grant no. 216593/Z/19/Z), Biotechnology and Biological Sciences Research Council (BBSRC) (MJ), University of Oxford Clarendon Fund and Merton College Tira Wannamethee Graduate Scholarship (MH), Medical Research Council (MRC) (JQ and JC-K), and the National Institute for Health and Care Research-Oxford Biomedical Research Centre (NIHR-BRC) (KX and JC-K). The views expressed are those of the authors and do not necessarily represent those of the Wellcome Trust, MRC or NIHR.

## Conflict of interest

The authors declare that the research was conducted in the absence of any commercial or financial relationships that could be construed as a potential conflict of interest.

## Publisher’s note

All claims expressed in this article are solely those of the authors and do not necessarily represent those of their affiliated organizations, or those of the publisher, the editors and the reviewers. Any product that may be evaluated in this article, or claim that may be made by its manufacturer, is not guaranteed or endorsed by the publisher.

## Footnotes

^1^ https://sph.uth.edu/retnet/
